# Tumor-derived apolipoprotein E confers resistance to temozolomide in pancreatic neuroendocrine tumors

**DOI:** 10.1038/s41419-025-08317-1

**Published:** 2025-12-13

**Authors:** Xin Lou, Yihua Shi, Yi Qin, Wuhu Zhang, Zeng Ye, Fei Wang, Yan Wang, Tian Ding, Desheng Jing, Guixiong Fan, Yue Zhang, Xuemin Chen, Xiaowu Xu, Xianjun Yu, Shunrong Ji, Junfeng Xu

**Affiliations:** 1 Center for Neuroendocrine Tumors, Fudan University Shanghai Cancer Center, Shanghai, 200032 China; 2 Department of Pancreatic Surgery, Fudan University Shanghai Cancer Center, Shanghai, 200032 China; 3 Department of Oncology, Shanghai Medical College, Fudan University, Shanghai, 200032 China; 4 Shanghai Pancreatic Cancer Institute, Shanghai, 200032 China; 5 Pancreatic Cancer Institute, Fudan University, Shanghai, 200032 China; 6 The First People’s Hospital of Changzhou, The Third Affiliated Hospital of Soochow University, Changzhou, China

**Keywords:** Neuroendocrine cancer, Oncogenes

## Abstract

Temozolomide (TMZ) is a first-class clinical drug for patients with pancreatic neuroendocrine tumors (pNETs). However, the therapeutic effects of TMZ are limited because of the chemoresistance of pNET cells, which has not been fully elucidated. Here, we demonstrate that the reprogramming of lipid metabolism regulates TMZ resistance in patients with pNETs. Via integrated multiomics sequencing, apolipoprotein E (APOE), which is a critical lipid carrier, was identified to be highly increased in the tissue and blood plasma of patients in the TMZ treatment group compared with those in the control group. Further mechanistic studies revealed that TMZ treatment promotes the expression and secretion of APOE, which binds to its surface receptor known as scavenger receptor class B member 1 (SCARB1), thus leading to increased uptake of exogenous lipids to remodel cellular lipid metabolism and activation of the homologous recombination repair (HRR) pathway to repair DNA damage via the β-catenin-BRCA1/2 axis. The interruption of APOE-mediated lipid uptake via a SCARB1 inhibitor named as block lipid transport-1 (BLT-1), suppressed TMZ-induced HRR activation and sensitized tumor cells to TMZ treatment in preclinical models, including PDCs, PDOs, and PDXs. In addition, APOE expression levels were shown to be positively correlated with BRCA1/2 expression in clinical specimens and online databases. This study reveals a new functional role of APOE that leads to chemoresistance in patient treatment. Our findings suggest the potential of combined administration of BLT-1 to overcome TMZ chemoresistance and improve treatments for patients with pNETs.

## Introduction

Pancreatic neuroendocrine neoplasms (pNENs) are the second most common type of pancreatic tumor [1–3]. More than 90% of pNENs are classified as pancreatic neuroendocrine tumors (pNETs), and most of them are nonfunctional tumors [4]. Due to a lack of early symptoms, more than half of patients are diagnosed with distant metastases, which are mainly observed in the liver [5, 6]. Temozolomide (TMZ) was the first class of clinical chemotherapeutic drug used for patients with aggressive pNETs [7]. However, due to the high heterogeneity of the tumor itself and the tumor microenvironment [8], only a small percentage of patients benefit from TMZ-based therapeutic modalities [9]. TMZ chemoresistance and cellular toxicity are still the main clinical problems that clinicians encounter during this treatment. Strategies to overcome chemoresistance and improve patient outcomes are of clinical importance.

TMZ is a small lipophilic alkylating drug that reacts with the purine bases of DNA to form methylated purines, such as O6‑methylguanine, thus leading to double-strand DNA breaks, which ultimately results in cell cycle arrest and cell death [10–12]. However, studies have shown that intrinsic and acquired TMZ resistance in cancer patients is responsible for their poor prognoses after treatment [13]. A variety of critical determinants can promote TMZ chemoresistance through multiple mechanisms [13–15]. In tumor cells, TMZ chemoresistance is mainly attributed to the activation of DNA damage repair pathways, including homologous recombination repair, nonhomologous end joining, mismatch repair, base excision repair, and O6-methylguanine DNA methyltransferase (MGMT)-mediated DNA methyl adduct removal [14]. Our previous study reported that the loss of MEN1 in pNETs can induce a high level of MGMT expression, which impairs the tumor cell response to TMZ via MEN1 deletion mutation-driven activation of the upstream pathway of MGMT [16]. This indicates that the intrinsic activation of signaling networks in tumors (to an extent) compensates for the disruption of MGMT after treatment with the MGMT-specific inhibitor O6-benzylguanine (O6-BG) in combination with TMZ, thus leading to unfavorable improvements in patient outcomes [17, 18]. Recent intensive studies have focused on the regulation of MGMT and the effects elicited by TMZ-derived metabolites [14, 19, 20]. However, whether pancreatic neuroendocrine tumor-associated metabolic reprogramming plays a role in TMZ chemoresistance remains unknown.

Tumor-associated metabolic reprogramming is a range of modifications of intra- and extracellular metabolites that are involved in tumorigenesis and development, such as lipid metabolism, glycolysis, oxidative phosphorylation, nucleotide metabolism and amino acid metabolism [21, 22]. These metabolites play important roles in cellular biosynthesis, tumor microenvironment remodeling, signal transduction, and gene expression and regulation, thus leading to the maintenance of tumor survival and growth [4, 23]. In mammalian cells, apolipoprotein E (APOE) serves as an important lipid transfer carrier that regulates cellular lipid uptake and metabolism by binding with lipids to form lipoproteins [24]. Studies have demonstrated that it plays a key role in the pathogenesis of Alzheimer’s disease and atherosclerosis [25]. Recent studies have shown that APOE can also participate in the regulation of tumor progression [26]. Some studies have reported that tumoral and stroma-derived APOE may function as an antiangiogenic and metastasis inhibitory protein that suppresses melanoma development [27], and APOE can also promote antitumor immunity by modulating myeloid immune cell populations and enhancing cytotoxic T lymphocyte responses [28]. In contrast, another study revealed that prostate cancer-derived APOE can bind with its receptor (trigger receptor expressed on myeloid cells 2 [TREM2]) on neutrophils and induce their senescence; moreover, APOE expression is associated with poor prognosis in prostate cancer patients [29], thus indicating its oncoprotein feature. However, its roles in the development of pNET and TMZ chemoresistance are still unclear.

In this study, we found that a high level of cholesterol significantly decreased the chemosensitivity of pNET cells to TMZ. TMZ-induced APOE was identified to play a critical role in the regulation of lipid metabolism and TMZ chemoresistance in tumors. Mechanistically, APOE can bind to its own receptor (SCARB1) in an autocrine manner, thus leading to the reprogramming of cellular lipid metabolism and the activation of the HR repair system via the β-catenin/TCF-BRCA1/2 axis, which confers TMZ chemoresistance. Disruption of APOE function via the SCARB1-specific inhibitor BLT-1 sensitized pancreatic neuroendocrine tumors to TMZ treatment. Our study reveals a previously unknown function of APOE in pNETs and offers a new combination modality for treating patients with TMZ-resistant pNETs.

## Materials and methods

### Clinical samples

The cohort 1 consisted of 28 drug-naïve patients and 12 patients with TMZ therapy who underwent surgical resection were monitored from February 2021 to August 2021, and the tissue specimens and corresponding 10 ml peripheral blood were collected. The cohort 2 consisted of 35 drug-naïve patients who underwent surgical resection from June 2023 to December 2023, with clinical details summarized in Table S1.

### Cell culture, cell transfection, and stable cell

The BON1 cell line was a gift from Professor Martyn Caplin (North West 32QG, Royal Free Hospital, Pound Street, London). The QGP1 cell line was purchased from the Cell Bank of the Research Biological Resources Preservation Center (JRCB, Osaka, Japan). BON1 and QGP1 were cultured in DMEM/Ham F12 and RPMI-1640 medium supplemented with 10% FBS, or 10% depletion lipid FBS (DLPS), or 10% DLPS with APOE (10 ng/ml), respectively. Plasmid or siRNA transfection was performed using lipofectamine and RNAiMax respectively. The short hairpin RNAs (shRNAs) targeting TCF3, APOE and SCARB1, and siRNA targeting the CTNNB1 were designed and synthesized by Genomeditech (Shanghai, China). The sequences of the shRNA or siRNAs were provided in Table S2. The coding sequencing of TCF3 and CTNNB1 were cloned into lentiviral pCDH-CMV-MCS-EF1-puro vector. After lentivirus infection of cancer cell, puromycin selection was applied for two weeks to obtain stable cells.

### Reagents and antibodies

Antibodies were obtained from the following sources, unless indicated otherwise: BRCA1 (Abclonal, A11549 and A11034), BRCA2 (Ablonal, A2435), β-catenin (Proteintech, 51067-2-AP), γH2AX (Abcam, ab81299), and β-actin (Abclonal, AC006). TMZ and LGK-974 were purchased from Selleck. BLT-1, APOE and ICG-001 were obtained from MCE. Wnt3a was purchased from Biotechne.

### In vitro drug treatment

BON1 and QGP1 were seeded in a 6-well plate and cultured in DMEM or DMEM/F12 supplement with 10% FBS 37 °C for 24 h. Cells were treated with 200 μM TMZ, 1 nM LGK-974, 100 nM BLT-1, 2 μM ICG-001, 10 ng/ml APOE and 10 ng/ml Wnt3a in culture medium for 24 h or 48 h. After treatment, cells were gently washed with cold PBS twice and then collected and stored at –80 °C. Western blot or RT-qPCR analysis were carried out.

### Proliferation assays

In total, 10,000 cells transfected with shNC, shAPOE or shSCARB1 were seeded in triplicate on 96-well plates. In the same way, 10,000 cells of each cell line were plated supplemented with either DMEM containing 10% FBS, or 10% delipidated FBS, or 10% delipidated FBS with cholesterol. Subsequently, the cells were counted every other day.

### RT-qPCR

Trizol® reagent (Invitrogen) was used to isolate total RNA as per manufacturer’s instructions. cDNA was synthesized from total RNA (2500 ng) using 10 µl 5× PrimeScript RT Master Mix (TaKaRa) in a 50 µl reaction mixture at 37 °C for 15 min and 85 °C for 5 s. PCR amplification was performed with the SYBR Green PCR master mixture (Qiagen), and the PCR amplified gene products were analyzed. mRNA expression levels were normalized to GAPDH endogenous controls and 2^-ΔΔ^ Ct values were used. Three biological replicates were performed. The sequences of the primers for RT-PCR were listed in Table S3.

### pNET patient tumor-derived cultures

pNETs PDC, PDO and PDX were used after obtaining patient information consent and approval from the Ethics Committee of Fudan University Cancer Center. Written informed consent from patients was obtained for the study. All procedures carried out in studies involving human participants are in accordance with international ethical guidelines for biomedical research involving human subjects. The pNETs PDC was built by our team as described earlier [30]. For PDO, 250 cells per 25 µl of growth factor-reduced Matrigel® (BD) were mixed and inoculated with N2 medium containing 10% R-reactive protein-1 CM, 100 µg/ml (Gibco), 1.25 mM acetylcysteine (Sigma), 10 mM niacinamide (Sigma), and 3 µM SB202190 (Sigma) were added. For PDX, fresh tissues were grafted into NSG mice. The tumor was then allowed to reach about 2000 mm [3]. The tumor volume was calculated as 0.5×length×width [2]. Tumor growth was closely monitored and when tumor volume reached 100mm [3], mice were randomized and divided into groups. The mice were randomly divided into four groups, which were treated with vehicle, BLT-1 (50 mg/kg/day) and TMZ (40 mg/kg/day) by oral gavage for 3 weeks, respectively.

### Mouse models and drug treatment

BALB/C-Nu mice (4–6 weeks old) were injected in each flank with BON1. Prior to injection, cells were grown in complete media (DMEM/F12 containing 10% FBS) in 15-cm dishes. For the experiments using a standard chow diet or high cholesterol diet, mice were fed the appropriate diet for 2 weeks before cell injection. Tumor growth was monitored with calipers every other day.

### High-fat diet (HFD) feeding protocol for nude mice

To model metabolic dysregulation, mice (8-week-old male athymic nude mice) are acclimated for 1 week on standard chow before randomization into HFD (60% kcal from fat; D12492) or matched control diet (10% kcal from fat; D12450B) groups. The HFD formulation contains 24% lard, 20% casein, 10% sucrose, and 1.25% cholesterol by weight, with vitamin/mineral supplementation. Mice are housed under controlled conditions (12-h light/dark cycle, 22 °C) with ad libitum access to food and water for 12–16 weeks. Body weight is monitored weekly, while fasting blood glucose and serum lipids (total cholesterol, triglycerides) are measured biweekly. Adjust feeding duration (8 weeks) to avoid excessive metabolic stress due to immune compromise. Post-experiment, tissues (liver, adipose, tumor) are harvested for downstream analysis of lipid accumulation, inflammatory markers, or tumor progression.

### Flow cytometry assay

Cells were harvested using cell dissociation buffer and resuspended in FACS buffer (PBS, 1% BSA), and flow cytometry was performed using CytoFLEX (Beckman Coulter). FITC channel was used to collect GFP signal, and raw data was analyzed by FlowJo version 10.8.1.

### Chromatin immunoprecipitation

Chromatin Immunoprecipitation (ChIP) was performed with the Millipore ChIP Kit with slight modification. Following sonication, lysates were precleared with ProteinA/G-Dynabeads for 2 h. Equal amounts of precleared lysates were incubated with IgG or specific antibodies overnight, following by precipitation with protein A/G-Dynabeads for 2 h. RT-PCR was performed to quantify the promoter occupancy.

### Western blot analysis

Cytoplastic cell lysates were prepared by NP-40 lysis buffer (Meilunbio, China) for immunoprecipitation experiments and RIPA lysis buffer (Beyotime, China) with inhibitors for other proteins. Nuclear proteins were isolated by a kit according to the modified protocols from the manufacturer (Thermo, USA). The cell protein lysates were extracted, denatured at 100 °C for 10 min, and separated by SDS-PAGE, then blotted onto polyvinylidene fluoride membranes. After blocking, the membranes were incubated with corresponding antibodies.

### ATP assay

Extracellular ATP concentration in BON1 supernatant was analyzed using an ATP Chemiluminescence Assay Kit (Cat: E-BC-F002, Elabscience) according to the manufacturer’s instructions.

### RNA sequencing

The RNA sample preparation, sequencing and data analysis were performed as previously reported [31, 32]. The genes with Log2fold change ≥1 or ≤–1 as well as FDR < 0.01 were considered upregulated or downregulated genes, respectively. The Gene Ontology and KEGG pathway analyses were performed for upregulated genes in BON1 cells according to the Bioconductor package of “ClusterProfiler” in R 3.5.2.

### LC-MS/MS-based proteomics

For LC-MS/MS-based proteomics, the cultured BON1 cells were divided into two groups and treated with TMZ and DMSO, respectively. The proteins of the two groups were extracted and identified by TMT labeling, high-performance liquid chromatography (HPLC), and quantitative proteomics based on mass spectrometry (MS).

### Lipidomic analysis

BON1 cells were treated with DMSO or 200 μM TMZ for 72 h, respectively. Cells were digested and collected. Cells were then frozen in liquid nitrogen and stored at –80 °C. The subsequent operations were provided by Shanghai Biotree Biotech. Briefly, LC-MS/MS analyses were performed using a UHPLC system (Vanquish, Thermo Fisher Scientifics), equipped with a Kinetex C18 column (2.1 × 100 mm, 1.7 μm, Phenomen). The QE mass spectrometer was used for its ability to acquire MS/MS spectra on data dependent acquisition mode in the control of the acquisition software (Xcalibur 4.0.27, Thermo).

### Genotyping of APOE variants

APOE genotyping was performed based on the analysis of two critical single-nucleotide polymorphisms (SNPs): rs429358 and rs7412. Genomic DNA was extracted from 35 pNET samples. The two SNPs were genotyped using TaqMan allelic discrimination assays (Applied Biosystems) on a real-time PCR system according to the manufacturer’s protocols. After amplification, endpoint fluorescence data were analyzed to assign genotypes at both loci.

### Gene sets enrichment analysis

We performed gene set enrichment analysis (GSEA) using “clusterProfiler” in R package to investigate the biological process difference. The Kyoto Encyclopedia of Genes and Genomes (KEGG) are exhibited by a GSEA plot. Patients-related gene set enrichment analysis for pNETs was carried out using publicly available gene expression data of 350 pNETs samples (accession number GSE43797, GSE73338, GSE73339, GSE117851).

### Confocal microscopy

5 × 10^5^ cells were fixed at room temperature (4% paraformaldehyde) for 20 min, washed with cold PBS, and permeated under RT (0.05%Triton X-100) for 10 min. The cells were then washed with cold PBS and continued for 1 h at 4 °C. The derived cells were incubated 4 °C overnight with primary antibody (1:100) in a sealed buffer at 4 °C, washed twice with cold PBS, and then rotated with secondary antibody (1:200) in a sealed buffer at RT for 1 h. After 30 min staining with DAPI, confocal microscopy (LSM510; Carl Zeiss, Germany) observed fluorescent signals and analyzed them using Zen software V3.1 (Carl Zess).

### Immunohistochemistry

pNETs tissue was rinsed with PBS, cut lengthwise, fixed with 10% neutral buffer formalin for 24 h, and buried in paraffin as Swiss rolls. For immunohistochemistry, 5 µm paraffin sections were dewaxed, rehydrated, and boiled in 10 mM sodium citrate (pH 6.0) solution for 25 min. Sections were then rinsed in TBS and enclosed at room temperature for 1 h with TBS containing 3%BSA and 0.1% Tween-20, then incubated overnight at 4 °C with primary antibody diluted in an enclosed buffer. On the second day, sections were rinsed in TBS and incubated at room temperature with 3%H2O2 for 15 min, then incubated at room temperature with goat anti-Rabbit IgG HRP (Abclonal, AS014) secondary antibodies diluted at 1:300 in a closed buffer for 1 h. Staining was observed with liquid DAB+ substrate color development system (Dako, K3468). Mean densities of IHC staining were determined by Image-J.

### Statistical analysis

The results are shown as a bar chart showing the Mean + SD, and a block chart showing the median and curve. In paired data, the normally distributed numeric variables were evaluated by paired *t* test, whereas non-normally distributed variables were analyzed by Wilcoxon rank-test. Pearson correlation coefficient R is used for correlation between two variables.

## Results

### High lipid metabolism confers resistance to TMZ in pNETs

**Fig. 1:**
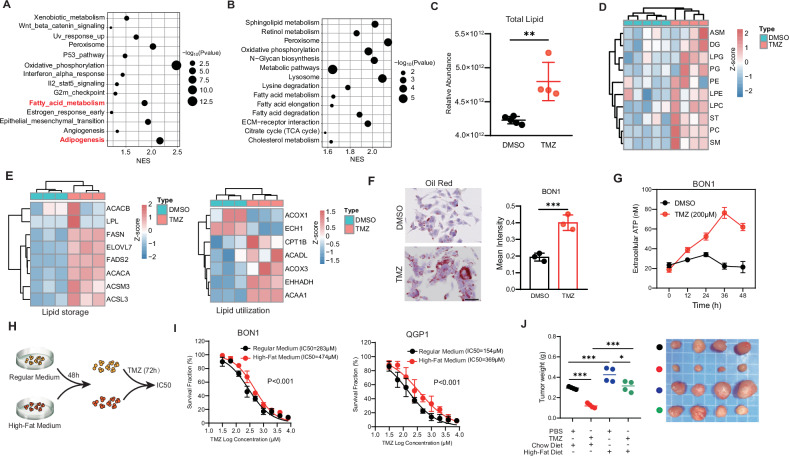
High lipid metabolism confer resistance to TMZ in pNETs. Enrichment analysis of hallmarks (**A**) and KEGG (**B**) pathways based on proteomic data in TMZ (200 μM)-treated BON10. Comparation of UPLC-MS lipidomics in TMZ (200 μM)-treated BON1, including the total lipid (**C**), and subclass of lipid (**D**). **E** Comparing key genes involved in lipid utilization and lipid storage based on transcriptomic data in TMZ (200 μM)-treated BON1. **F** The effects of TMZ (200 μM) on cellular lipid droplets in BON1. Scale bar, 50 μm. **G** The impact of different exposed period of TMZ on the extracellular ATP. **H** Experimental design for I. The high-fat cell model was induced with cholesterol (10 μg/mL) for 48 h. **I** The cytotoxicity assay curves of TMZ in BON1 treated with high-fat or DMSO. **J** Representative photographs of TMZ-treated BON1 grown in mice with a standard chow diet or cholesterol diet.

To investigate the impact of TMZ on the biological function of pNETs, BON1 cells were treated with either TMZ or DMSO for 3 days. Subsequently, the cells were collected for transcription sequencing and proteomics analysis. In the functional enrichment analysis of the proteome, we observed that TMZ induced a significant increase in the metabolic levels of tumor cells, particularly in lipid metabolism. Hallmarks enrichment revealed that TMZ induced the ADIPOGENESIS and the FATTY_ACID_METABOLISM in pNET cells based on proteomics analysis (Fig. 1A). Similar results were demonstrated through KEGG analysis (Fig. 1B). Consistent with proteomics data, the RNA-seq in TMZ-treated BON1 also showed higher lipid metabolism level (Fig. S1A). Subsequent lipidomic analysis showed that TMZ led to an increase in total lipid levels in BON1 (Fig. 1C), especially in PG, PE, SM, among others (Fig. 1D). We then investigated the genes related to lipid metabolism, including lipid storage and lipid utilization. Interestingly, we find the most of key genes involved in lipid storage and lipid utilization both showed remarkably higher expression in TMZ-treated cells relative to DMSO-treated (Fig. 1E). We further validated the expression of key genes involved in lipid storage and lipid utilization using RT-PCR assays. Consistent with RNA-seq data, the key genes including ACACA, ECH1, CPT1B and ACADL were dramatically higher in TMZ-treated BON1 relative to DMSO-treated. To further assess intracellular lipid level, we carried out the Nile Red staining. We found that lipid droplets were dramatically increased in TMZ-treated relative to DMSO-treated BON1 (Fig. 1F). We next examined the activity of lipid utilization by measuring the extracellular ATP, and a significant increase in extracellular ATP levels was observed (Fig. 1G). The exacerbation of both fat storage and utilization can be attributed to TMZ-induced increase in external uptake of fat by tumors. In other word, the increased fat storage and utilization induced by TMZ may lead to drug resistance and survival of tumor cells. Therefore, we induced high-fat BON1 and QGP1 cells with cholesterol (10 μg/mL) and then treated them with TMZ (Fig. 1H). It was observed that the IC50 of the high-fat BON1 and QGP1 cells did increase (Fig. 1I). Furthermore, high-fat feeding in mice yielded similar results, demonstrating that high-fat diet mice did indeed confer resistance to TMZ (Fig. 1J).

### TMZ promotes the secretion of APOE by pNETs to remodel lipid metabolism

**Fig. 2:**
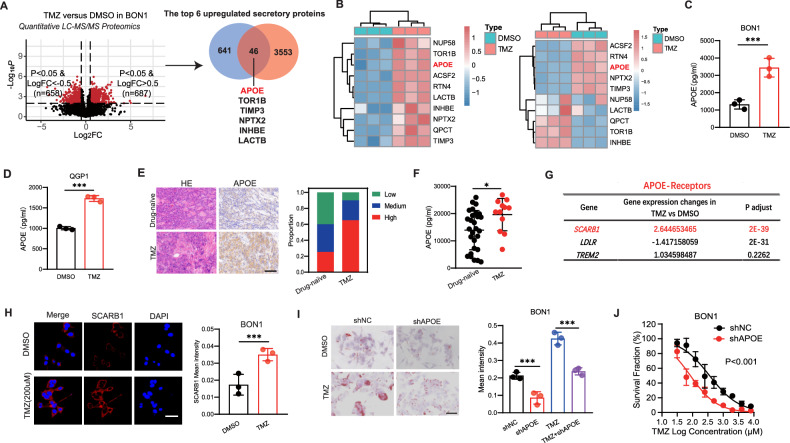
Temozolomide promotes the secretion of APOE by pNETs to reshape lipid metabolism. **A** Intersection of the upregulated proteins in TMZ-treated BON1 with all secreted proteins recorded in the website (https://www.proteinatlas.org/) and present of the top 6 secreted proteins in TMZ-treated BON1 based on Log2fold change. **B** Heatmaps were generated to show the mRNA and protein expression of these top 6 secreted proteins in the DMSO and TMZ-treated cells from RNA-seq data (right) and Proteomics data (left), respectively. **C** Elisa results revealed that APOE level in the supernatant of the TMZ-treated BON1. **D** Elisa results revealed that APOE level in the supernatant of the TMZ-treated QGP1. **E** Representative images of IHC staining for APOE expression in pNET patients with TMZ therapy and drug-naïve patients from cohort 1. Score <3, low; Score ≥3 and <9, median; Score ≥9, high. Scale bar, 100 μm. **F** ELISA analysis indicated APOE levels of plasma samples in cohort 1. **G** APOE-receptors expression in TMZ-treated BON1 from RNA-seq data. **H** The expression of APOE-receptor SCARB1 on the TMZ-treated BON1 using cell fluorescence. Scale bar, 50 μm. **I** Impact of APOE knockdown on the change of TMZ-induced lipid droplets in BON1. Scale bar, 50 μm. **J** Impact of APOE knockdown on BON1 sensitivity to TMZ. Data in the graph are shown as the means ± SD from three independent experiment. *P < 0.05, **P < 0.01, ***P < 0.001, ns: no significance.

Due to the fact that TMZ leads to a significant increase in both lipid utilization and storage, TMZ caused tumors to uptake large amounts of exogenous lipids. Previous studies had shown that tumors contributed to chemotherapy resistance in an autocrine manner [33, 34]. Therefore, we speculated whether pNETs might also exhibit autocrine mechanisms that lead to TMZ resistance. In order to identify the key secreted proteins, we intersected the upregulated proteins in TMZ-treated BON1 based on proteomics with all secreted proteins recorded on the website (https://www.proteinatlas.org/), and presented the top 6 secreted proteins: APOE, TOR1B, TIMP, NPTX2, INHBE, and LACTB (Fig. 2A). Heatmaps were generated to show the mRNA and protein expression of these top 6 secreted proteins in the DMSO and TMZ-treated cells, respectively (Fig. 2B). Because there were numerous literature reports on the association between APOE and cellular metabolism, particularly lipid metabolism, it was consistent that we had observed a significant increase in lipid metabolism levels. Through RT-qPCR, it was found that APOE expression in TMZ-treated pNET cells was significantly higher than that in DMSO-treated cells (Fig. S2A). Additionally, Elisa results revealed that APOE level in the supernatant of TMZ-treated pNET cells was significantly higher in BON1 and QGP1 (Fig. 2C, D). IHC staining confirmed APOE expression was higher in pNET patients with TMZ therapy than drug-naïve patients from cohort 1 (Fig. 2E). Then, ELISA analysis indicated APOE levels in plasma of patients with TMZ therapy present higher than drug-naïve patients from cohort 1 (Fig. 2F). Many studies had reported that APOE has three corresponding receptors including SCARB1, LDLR, and TREM2 [29, 35]. Through transcriptome data, we had observed that the SCARB1 was significantly upregulated in TMZ-treated BON1 (Fig. 2G). Then, RT-qPCR revealed that APOE-receptor SCARB1 level was elevated in TMZ-treated BON1 and QGP1 (Fig. S2B). We further observed TMZ could increase the expression of APOE-receptor SCARB1 receptors based on cell fluorescence (Fig. 2H). We further utilized the SCARB1 inhibitor BLT-1, and observed a significant increase sensitivity of BON1 and QGP1 to TMZ (Fig. S2C). Subsequently, upon separately knocking down APOE and SCRRB1 in BON1, we observed a significant reduction in the number of lipid droplets induced by TMZ, as well as an increase in sensitivity of the tumor to TMZ (Fig. 2I-J, Fig. S2D-E).

### APOE decreases TMZ sensitivity by Homologous recombination pathway

**Fig. 3:**
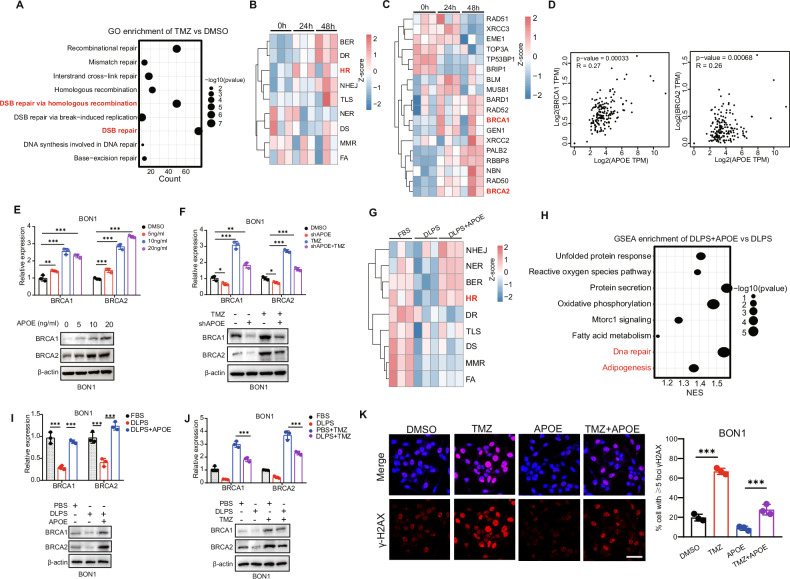
APOE decreases TMZ sensitivity by Homologous recombination pathway. **A** GO functional enrichment analysis in TMZ-treated cells reveals the activation of various DNA damage pathways. **B** ssGSEA analysis indicates the score of DNA damage pathways in APOE (10 ng/ml)-treated cells based on transcriptomic data at different time points. **C** The expression of different genes related to the Homologous recombination (HR) pathway in APOE (10 ng/ml)-treated cells. **D** Based on GEPIA and TCGA data, the correlation between APOE expression and the HR genes BRCA1 and BRCA2 in pancreatic tissues. **E** Western blotting and RT-PCR experiments revealed that APOE enhances the expression of the HR genes BRCA1/2. **F** Knocking down APOE decreased expression of the HR genes BRCA1/2 induced by TMZ. **G** Comparison of various DNA damage repair pathway scores among the 10%FBS culture medium, 10% DLPS culture medium, and APOE (10 ng/ml)+10% DLPS culture medium, based on the transcriptomic data (48 h). **H** Comparison of functional enrichment analysis between the APOE(10 ng/ml)+10% DLPS culture medium and 10% DLPS culture medium group. **I** Comparison of various DNA damage repair pathway genes among the 10% FBS culture medium, 10% DLPS culture medium, and APOE(10 ng/ml)+10% DLPS culture medium cell. **J** Comparison of various DNA damage repair pathway genes among the 10% FBS culture medium, 10% DLPS culture medium, 10% FBS culture medium + TMZ, and 10% DLPS culture medium + TMZ cell. **K** Cell fluorescence response of APOE reduced the proportion of TMZ-induced DNA damage cells. Data in the graph are shown as the means ± SD from three independent experiment. *P < 0.05, **P < 0.01, ***P < 0.001, ns: no significance.

Previous studies had indicated a link between chemotherapy resistance and DNA damage repair [36, 37], but the specific mechanism underlying resistance to TMZ in tumors remains unclear. DNA damage repair genes in the same pathway often work in concert to repair specific types of DNA damage [38]. Base excision repair (BER), nucleotide excision repair (NER), and the direct damage reversal/repair (DR) pathways repair DNA base damage, while mismatch repair (MMR) corrects base mispairs and small loops often found in repetitive sequence DNA. Homologous recombination (HR), non-homologous end joining (NHEJ), the Fanconi anemia (FA) pathway, and translesion DNA synthesis (TLS) act alone or together to repair DNA strand breaks and complex events like interstrand crosslinks [38, 39]. Based on transcriptional of TMZ-treated BON1, enrichment analysis revealed that TMZ-induced DNA damage repair primarily relies on HR related to double-strand break (DSB) repair in pNET cells (Fig. 3A). In order to investigate whether APOE induces HR repair, we administered APOE treatment to BON1 cells for different time periods. Based on the gene signatures of different DNA repair from the study [38], we conducted ssGSEA analysis and found that APOE indeed activates HR repair at 24 h and activates most DNA repair pathways at 48 h (Fig. 3B). We then used a heatmap to demonstrate the differential activation of HR-related genes by APOE at different time points (Fig. 3C). Numerous studies have reported the critical roles of BRCA1/2 in HR repair. Subsequently, we utilized publicly available data from GEPIA and TCGA to uncover a statistically significant correlation between the expression of APOE and BRCA1/2 in pancreatic tissues (Fig. 3D). Then, we also found there was correlation between APOE and BRCA1/2 based on transcriptomic data from cohort 2 (Fig. S3A). We further validated through Western blotting and RT-PCR that higher APOE concentration increase the expression of BRCA1/2 (Fig. 3E, Fig. S3B). Additionally, we observed that knockdown of APOE effectively reduces TMZ-induced HR repair (Fig. 3F, Fig. S3C). To demonstrate that APOE influences cellular DNA repair through lipid metabolism, we utilized 10%DLBS medium in comparison to 10%FBS medium and observed a significant decrease in DNA repair capacity (Fig. 3G). Subsequently, upon supplementation of APOE into DLBS medium, we found partial activation of DNA damage pathways, including the HR repair pathway (Fig. 3G). The functional enrichment analysis of hallmark revealed that APOE indeed enhances ADIPOGENESIS and DNA_REPAIR (Fig. 3H). RT-PCR confirmed our aforementioned findings that APOE can increase HR repair efficiency of DSBs in 10%DLBS (Fig. 3I). Similarly, low lipid levels can decrease the HR repair efficiency of DNA induced by TMZ (Fig. 3J). The cellular fluorescence response of APOE decreased the proportion of DNA-damaged cells induced by TMZ (Fig. 3K).

### APOE activated Wnt signal pathway and subsequent Wnt-induced DNA repair

**Fig. 4:**
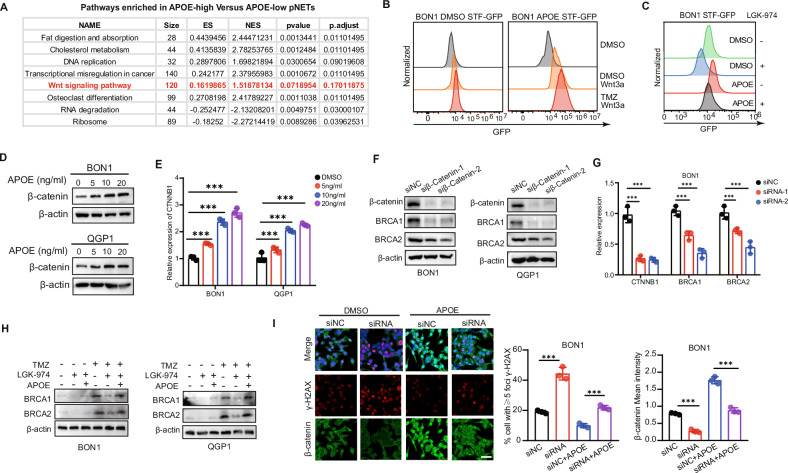
APOE activated Wnt signal pathway and subsequent Wnt-induced DNA repair. **A** GSEA analysis revealed enrichment of pathways in APOE-high relative to APOE-low group based on 350 pNETs patients (GSE43797, GSE73338, GSE73339, GSE117851). The patients were divided into APOE-high and APOE-low groups based on the median expression value of APOE. **B** APOE (10 ng/ml) promotes Wnt3a-induced STF-GFP reporter in BON1. **C** LGK-974 blocks the high expression of STF-GFP induced by APOE (10 ng/ml) in BON1. **D** WB showed APOE increase β-catenin accumulation in BON1 and QGP1. **E** PCR showed APOE increase β-catenin accumulation in BON1. **F** WB showed knockdown of β-catenin by independent siRNAs inhibits HR genes BRCA1/2 in BON1 and QGP1. **G** RT-PCR showed knockdown of β-catenin by independent siRNAs inhibits HR genes BRCA1/2 in BON1. **H** LGK-974 inhibited the HR genes BRCA1/2 induced by TMZ, which is reversed by APOE in BON1 and QGP1. **I** APOE increased nuclear localization of β-catenin, and decreased the DNA damage induced by siβ-catenin in BON1. Scale bar, 50 μm. Data in the graph are shown as the means ± SD from three independent experiment. *P < 0.05, **P < 0.01, ***P < 0.001, ns: no significance.

We used 350 pNETs samples (GSE43797, GSE73338, GSE73339, GSE117851) to characterize pNET patients with high expression of APOE relative to those with low expression. GSEA analysis revealed enrichment of pathways in high APOE patients related to activation of Fat digestion and absorption, Osteoclast differentiation and Wnt signaling pathway (Fig. 4A). Previous studies have reported that the WNT pathway can activate DNA damage repair [40, 41], however, the specific mechanisms by which the Wnt pathway is involved in different DNA repair pathways remain unknown. We found that APOE can activate the WNT pathway. APOE promoted Wnt3a or TMZ induced STF-GFP expression (Fig. 4B). Treatment with the Wnt inhibitor LGK-974 completely suppressed STF-GFP expression induced by APOE (Fig. 4C). Western blot and RT-PCR showed that APOE could increase the expression of β-catenin (Fig. 4D, E, Fig. S4A). Then, we observed that knocking down CTNNB1 results in decreased expression of HR-related genes (Fig. 4F-G, Fig. S4B). Interestingly, we found that Wnt pathway inhibitors can reduce DNA repair induced by TMZ, but this diminished DNA repair capability can be reversed by APOE (Fig. 4H). Similarly, cell fluorescence revealed that APOE indeed can reverse the proportion of cells with DNA damage induced by siβ-catenin (Fig. 4I).

### APOE transcriptionally promoted HR pathways genes via TCF3

**Fig. 5:**
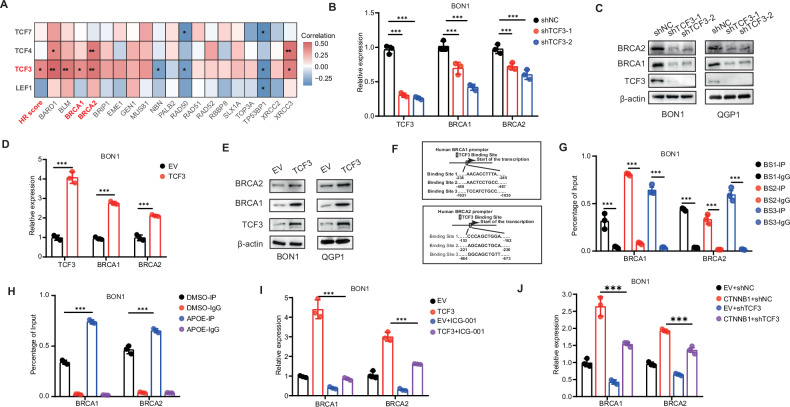
APOE transcriptionally promoted HR genes via TCF3. **A** The correlation analysis was determined by a two-tailed nonparametric Spearman correlation analysis between TCF/LEF in Wnt pathways with HR score or HR genes based on the transcriptomic data of cohort 2. **B**, **C** mRNA and protein expression of HR genes BCRA1/2 expression after knockdown of TCF3. **D**, **E** mRNA and protein expression of HR genes BRCA1/2 in cells expressing TCF3 plasmid. **F** Predicting three TCF3 binding site in human BRCA1and BRCA2 promoter through JASPAR Website, respectively. **G** The ChIP-PCR experiments were conducted to verify the binding ability of TCF3 to the three binding sites of BRCA1 and BRCA2, respectively. **H** The ChIP-PCR experiments investigated the impact of APOE on the transcriptional activation of TCF for BRCA1/2. **I** RT-qPCR analysis of BRCA1/2 expression in BON1 following treatment with ICG-001 and TCF3 plasmid in BON1. **J** mRNA expression of BRCA1/2 in shTCF3 BON1 with or without transfection of the CTNNB1 plasmid in BON1. Data in the graph are shown as the means ± SD from three independent experiment. *P < 0.05, **P < 0.01, ***P < 0.001, ns: no significance.

The TCF/LEF family of transcription factors are core members of the WNT signaling pathway, including TCF1, LEF1, TCF3, and TCF4. Therefore, we explored the correlation between TCF/LEF transcription factors and genes involved in the HR pathway. Interestingly, we found that the TCF3 transcription factor was positively correlated with the HR pathway and key HR genes BRCA1/2 based on cohort 2 (Fig. 5A). These findings suggested that BRCA1/2 might be a downstream effector of TCF3-mediated biological functions, which leads us to speculate whether the Wnt pathway influences the HR repair pathway through TCF3 transcriptional activation. After knocking down TCF3, RT-PCR and Western blot analyses showed a decrease in the expression of BRCA1/2 (Fig. 5B, C). To validate our results, we overexpressed TCF3 and observed an expected increase in the expression of BRCA1/2 (Fig. 5D, E). Subsequently, we individually designed potential binding sites for TCF3 on the promoters of BRCA1 and BRCA2, respectively (Fig. 5F). Through ChIP experiments, we discovered that TCF3 could activate the transcription levels of BRCA1/2 (Fig. 5G). Subsequently, we found that APOE was found to activate HR genes BRCA1/2 through transcriptional activation by TCF3 (Fig. 5H). ICG-001, a β-Catenin/TCF mediated transcriptional inhibitor, had been reported by lots of studies [42, 43]. We also found that the induction of BRCA1/2 expression by TCF3 was abrogated by ICG-001 (Fig. 5I). Furthermore, TCF3 depletion abolished the induction of BRCA1/2 expression by β-catenin (Fig. 5J).

### The SCARB1 inhibitor BLT-1 increased TMZ sensitivity in pre-clinical models

**Fig. 6:**
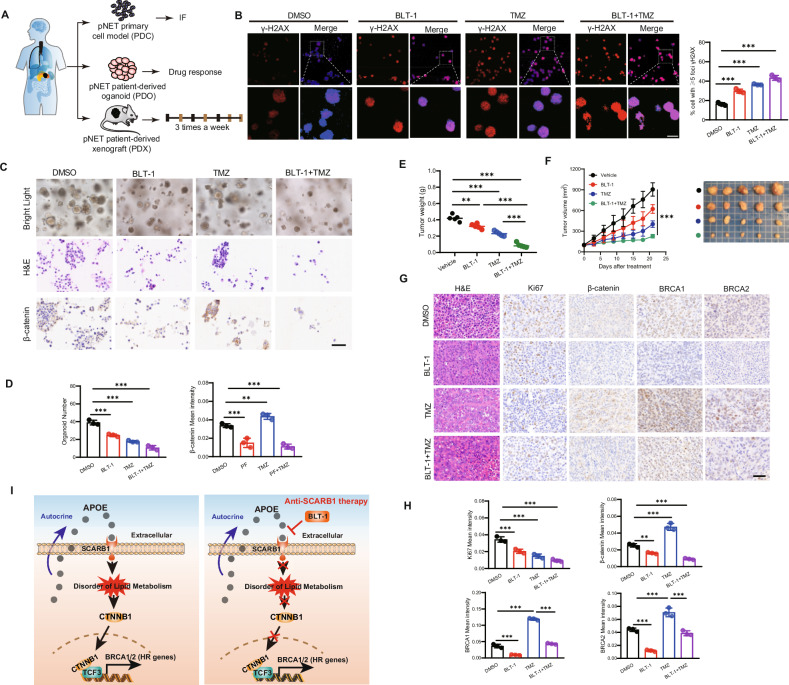
The SCARB1 inhibitor BLT-1 increased TMZ sensitivity in pre-clinical models. **A** Schematic of TMZ and BLT-1 treatment using PDCs, PDOs and PDXs from a patient. **B** Confocal laser scanning microscopy and quantification also revealed an increased proportion of γH2AX foci in PDCs after 48 h treatment. Scale bar, 10 μm. **C**, **D** Both TMZ treatment alone and BLT-1 + TMZ cotreatment effectively reduced the growth of PDOs after 72 h treatment. Confocal images β-catenin in organoid co-treated with TMZ and BLT-1 and quantification of the mean intensity. Scale bar, 50 μm. **E**, **F** Effect of TMZ treatment alone and BLT-1 + TMZ in PDX. Tumor weight, Tumor growth curves, and Representative graphs of tumors. **G**, **H** IHC staining and quantification of PDX showed that the higher expression of β-catenin, and BRCA1/2 induced by TMZ was reversed by BLT-1 + TMZ. Scale bar, 50 μm. **I** Schematic diagram of the principle of this work. TMZ induces the self-secretion of APOE by tumor cells, leading to dysregulation of lipid metabolism and activation of the Wnt pathway, as well as subsequent DNA damage repair. Mechanistically, the transcription factor TCF3 on the Wnt pathway activates the HR genes BRCA1/2. Data in the graph are shown as the means ± SD from three independent experiment. *P < 0.05, **P < 0.01, ***P < 0.001, ns: no significance.

To investigate the efficacy of co-treatment with the SCARB1 inhibitor BLT-1 and TMZ on tumor recurrence after TMZ treatment, we used PDC, PDO, and PDX from the same patient (Fig. 6A). This specific PDC was previously validated in our earlier study [30]. IHC staining of PDO and PDX samples confirmed a higher positive rate of neuroendocrine tumor markers CGA and SYN (Fig. S5A-B). The combination of SCARB1 inhibitor BLT-1 with TMZ reduced resistance to TMZ and confocal laser scanning microscopy revealed an increased proportion of γH2AX foci in PDCs (Fig. 6B). Both TMZ treatment alone and BLT-1 + TMZ co-treatment effectively decreased the growth of PDOs, with the BLT-1 reducing the expression of β-catenin induced by TMZ (Fig. 6C, D). PDXs showed significantly greater sensitivity to TMZ + BLT-1 co-treatment compared to TMZ treatment alone (Fig. 6E, F). IHC staining of tumors demonstrated that the elevated expression of β-catenin and HR genes BRCA1/2 induced by TMZ was reversed by BLT-1 (Fig. 6G, H). Overall, TMZ induces the autocrine of APOE by tumor cells, leading to dysregulation of lipid metabolism and activation of the Wnt pathway, and subsequent DNA damage repair. Mechanistically, the transcription factor TCF3 on the Wnt pathway activates the HR pathway genes BRCA1/2, thereby reducing the sensitivity to TMZ (Fig. 6I).

## Discussion

In our study, we discovered an intriguing phenomenon in which the administration of TMZ resulted in increased secretion of APOE, which was coupled with the upregulation of its receptor SCARB1. Further investigations revealed that APOE (through an autocrine pathway) plays a significant role in lipid metabolism and Wnt signal activation, thus ultimately leading to DNA damage repair and TMZ resistance. Although the relationship between APOE and chemotherapy remains poorly investigated, there is substantive evidence linking APOE to cognitive impairment in patients undergoing chemotherapy [44, 45]. The increased secretion of APOE induced by tumor chemotherapy results in brain cell aging, the establishment of a proinflammatory milieu, and disturbances in normal brain communication [44, 46]. Previous studies have reported an association between APOE and tumor progression in solid tumors [47, 48]. Interestingly, in TMZ-induced cells, the levels of genes involved in lipid metabolism (particularly lipid metabolism) were significantly elevated. Via functional enrichment methods, we discovered that TMZ leads to increased lipid storage and utilization in tumor cells, thus suggesting that TMZ enhances extracellular lipid uptake by tumor cells. Tumor-derived APOE, which is a critical lipoprotein, appears to play a key role in this process, which explains the increase in cellular lipid uptake. In our study, we demonstrated that TMZ stimulates tumor cells to secrete APOE, which then interacts with increased SCARB1 to initiate a series of cascade reactions. Notably, APOE in the tumor microenvironment is not solely derived from tumor cells, and its target cells extend beyond tumor cells alone [49]. Although our study did not explore the specific mechanism through which TMZ influences APOE secretion, this scenario remains a crucial area for future investigations.

Furthermore, the knockdown of APOE significantly decreased the expression of CTNNB1 and TCF3. Additionally, the targeting of APOE with shRNA resulted in reduced expression of CTNNB1 and TCF3 at both the mRNA and protein levels. TCF3, which is a crucial transcription factor in the Wnt pathway, modulates various downstream genes involved in tumor differentiation [50, 51]. This finding prompted us to investigate whether TCF3 functions as a transcription factor to regulate DNA damage repair genes. Via RT-qPCR and ChIP-PCR, we demonstrated that TCF3 can indeed transcriptionally regulate the HR genes BRCA1/2. Based on these findings, we propose that the APOE-CTNNB1/TCF3 axis can activate DNA damage repair. Hence, the inhibition of this axis may be important for suppressing tumor proliferation and the drug response.

APOE exhibits multifaceted oncogenic properties across malignancies. In breast cancer, APOE overexpression activates PI3K/Akt signaling to drive tumor growth and metastasis [52]. studies demonstrate APOE-mediated angiogenesis via VEGF upregulation in bladder cancer [53], while lung cancer research reveals its EMT-inducing capacity through ZEB1/vimentin axis activation [54]. These findings position APOE as a central regulator of proliferation, vascularization, and invasion. In addition, the study reported that APOE knockdown in glioblastoma cells attenuated tumor migration/invasion while reprogramming cytokine secretion—elevating antitumor cytokines (IL-6/IL-12/TNF-α) and suppressing immunosuppressive CCL5/TGF-β. This shift disrupted the CCL5/CCR5 axis, impairing M2 macrophage polarization and reducing protumor immune suppression [55]. APOE suppresses ferroptosis in papillary thyroid carcinoma (PTC) via the PI3K/AKT1 pathway, elevating GPX4/FTH1 expression while reducing Fe²⁺ accumulation. This ferroptosis inhibition drives M2 macrophage polarization, which enhances tumor cell invasion and migration [56]. These findings demonstrate that APOE functions as a pivotal molecular regulator in tumor progression and immune microenvironment.

MGMT is a critical enzyme in direct reversal repair (DR), which refers to the process of directly repairing damage via a single enzyme without involving DNA base excision or synthesis [57]. However, in addition to DR dependent on MGMT, TMZ also causes single- and double-strand DNA breaks and activates various downstream repair mechanisms, including BER, MMR, and HR [58, 59]. In our study, we addressed two key questions. First, we investigated which DNA repair mechanism is primarily triggered by TMZ; and second, we examined the mechanism by which TMZ induces this repair mechanism. In our study, we observed that TMZ-induced HR-mediated double-strand and exogenous APOE can indeed activate the HR pathway. Interestingly, when we interfered with the downstream effects triggered by APOE, we observed an increase in TMZ sensitivity. However, in terms of mechanistic explorations, we focused on HR repair, including the activation of the CTNNB1/TCF3 pathway by APOE-SCARB1, which leads to HR repair.

Our findings demonstrate a novel role for APOE in regulating the Wnt/β-catenin pathway and enhancing BRCA1/2-mediated DNA repair mechanisms in pNETs. Although all three APOE isoforms (E2, E3, and E4) were capable of activating this pathway, evidenced by increased β-catenin, BRCA1/2 expression, reduced γ-H2AX foci, and enhanced TCF3 promoter binding, the APOE3 isoform consistently elicited the most robust response (Fig. S6). This is particularly interesting given that the majority (85%) of the clinical pNET samples analyzed were homozygous for APOE3 (Table S4), suggesting a potential biological preference or heightened sensitivity in pNET contexts.

At last, we proposed the idea of intervening in this signaling axis via the SCARB1 receptor inhibitor BLT-1, which significantly enhances the sensitivity of cells to TMZ. Nevertheless, we must recognize that the inhibition of APOE-SCARB1 suppressed both downstream DNA repair pathways (including HR) and other pathways, such as DR, as we observed that APOE was able to activate repair pathways beyond HR. To determine whether CTNNB1/TCF3 can activate other DNA damage pathways, further investigations are needed. BLT-1 currently resides in early-phase clinical development with demonstrated preclinical efficacy in disrupting APOE/SCARB1-mediated tumor progression pathways. Its combination with PARP inhibitors (PARPi) shows compelling mechanistic synergy: By blocking cholesterol transport through SCARB1 inhibition, BLT-1 induces metabolic stress that depletes lipid stores essential for DNA damage repair, thereby potentiating PARPi-induced synthetic lethality in homologous repair-deficient tumors. This approach is particularly promising for APOE-overexpressing cancers (where our cohort data show association with tumor relapse), as it simultaneously targets resistance mechanisms while exploiting BRCA-deficient vulnerabilities.

## Supplementary information

Figure S1-S6

Table S1

Table S2

Table S3

Table S4

## Data Availability

The datasets used and/or analysed during this study are available from the corresponding author on reasonable request.

## References

[1] Dasari A, Shen C, Halperin D, Zhao B, Zhou S, Xu Y, et al. Trends in the incidence, prevalence, and survival outcomes in patients with neuroendocrine tumors in the United States. JAMA Oncol. 2017;3:1335–42. 28448665 10.1001/jamaoncol.2017.0589 PMC5824320

[2] Xu J, Ye Z, Zhuo Q, Gao H, Qin Y, Lou X, et al. MEN1 degradation induced by neddylation and the CUL4B-DCAF7 axis promotes pancreatic neuroendocrine tumor progression. Cancer Res. 2023;83:2226–47. 36939378 10.1158/0008-5472.CAN-22-3599

[3] Cives M, Strosberg JR. Gastroenteropancreatic neuroendocrine tumors. CA: A Cancer J Clin. 2018;68:471–87. 10.3322/caac.21493 30295930

[4] Xu J, Zhang W, Lou X, Ye Z, Qin Y, Chen J, et al. Recent research hotspots in sequencing and the pancreatic neuroendocrine tumor microenvironment. Cancer Biol Med. 2023;20:721–7. 37771139 10.20892/j.issn.2095-3941.2023.0284 PMC10618947

[5] Partelli S, Gaujoux S, Boninsegna L, Cherif R, Crippa S, Couvelard A, et al. Pattern and clinical predictors of lymph node involvement in nonfunctioning pancreatic neuroendocrine tumors (NF-PanNETs). JAMA Surg. 2013;148:932–9. 23986355 10.1001/jamasurg.2013.3376

[6] Touzios JG, Kiely JM, Pitt SC, Rilling WS, Quebbeman EJ, Wilson SD, et al. Neuroendocrine hepatic metastases: does aggressive management improve survival? Ann Surg. 2005;241:776–83. 15849513 10.1097/01.sla.0000161981.58631.ab PMC1357132

[7] Douangprachanh S, Joo HJ, Park HM, Han N, Jang HY, Koh YH, et al. Capecitabine and temozolomide for metastatic intermediate to high-grade pancreatic neuroendocrine neoplasm: a single center experience. Korean J Intern Med. 2022;37:1216–22. 36375489 10.3904/kjim.2022.100 PMC9666252

[8] Ye Z, Li Q, Hu Y, Hu H, Xu J, Guo M, et al. The stromal microenvironment endows pancreatic neuroendocrine tumors with spatially specific invasive and metastatic phenotypes. Cancer Lett. 2024;588:216769. 38438098 10.1016/j.canlet.2024.216769

[9] Zhang WH, Xu JF, Hu YH, Qin Y, Chen J, Yu XJ, et al. The surgical and therapeutic activities of non-functional pancreatic neuroendocrine tumors at a high-volume institution. Cancers. 2023;15:1955. 37046616 10.3390/cancers15071955 PMC10093673

[10] Jiang J, Xu J, Ji S, Yu X, Chen J. Unraveling the mysteries of MGMT: Implications for neuroendocrine tumors. Biochim et Biophys Acta Rev Cancer. 2024;1879:189184. 10.1016/j.bbcan.2024.189184 39303858

[11] Esteller M, Garcia-Foncillas J, Andion E, Goodman SN, Hidalgo OF, Vanaclocha V, et al. Inactivation of the DNA-repair gene MGMT and the clinical response of gliomas to alkylating agents. N Engl J Med. 2000;343:1350–4. 11070098 10.1056/NEJM200011093431901

[12] Hegi ME, Diserens AC, Gorlia T, Hamou MF, de Tribolet N, Weller M, et al. MGMT gene silencing and benefit from temozolomide in glioblastoma. N Engl J Med. 2005;352:997–1003. 15758010 10.1056/NEJMoa043331

[13] Kunz PL, Graham NT, Catalano PJ, Nimeiri HS, Fisher GA, Longacre TA, et al. Randomized study of temozolomide or temozolomide and capecitabine in patients with advanced pancreatic neuroendocrine tumors (ECOG-ACRIN E2211). J Clin Oncol : J Am Soc Clin Oncol. 2023;41:1359–69. 10.1200/JCO.22.01013 PMC9995105 36260828

[14] Yin J, Wang X, Ge X, Ding F, Shi Z, Ge Z, et al. Hypoxanthine phosphoribosyl transferase 1 metabolizes temozolomide to activate AMPK for driving chemoresistance of glioblastomas. Nat Commun. 2023;14:5913. 37737247 10.1038/s41467-023-41663-2 PMC10516874

[15] Rajabpour A, Rajaei F, Teimoori-Toolabi L. Molecular alterations contributing to pancreatic cancer chemoresistance. Pancreatol : J Int Assoc Pancreatol (IAP) [et al]. 2017;17:310–20. 10.1016/j.pan.2016.12.013 28065383

[16] Xu J, Lou X, Wang F, Zhang W, Xu X, Ye Z, et al. MEN1 Deficiency-Driven Activation of the beta-Catenin-MGMT Axis Promotes Pancreatic Neuroendocrine Tumor Growth and Confers Temozolomide Resistance. Adv Sci (Weinh). 2024;11:e2308417. 39041891 10.1002/advs.202308417 PMC11425246

[17] Quinn JA, Desjardins A, Weingart J, Brem H, Dolan ME, Delaney SM, et al. Phase I trial of temozolomide plus O6-benzylguanine for patients with recurrent or progressive malignant glioma. J Clin Oncol. 2005;23:7178–87. 16192602 10.1200/JCO.2005.06.502

[18] Quinn JA, Jiang SX, Reardon DA, Desjardins A, Vredenburgh JJ, Rich JN, et al. Phase II trial of temozolomide plus o6-benzylguanine in adults with recurrent, temozolomide-resistant malignant glioma. J Clin Oncol. 2009;27:1262–7. 19204199 10.1200/JCO.2008.18.8417 PMC2667825

[19] Chen X, Zhang M, Gan H, Wang H, Lee JH, Fang D, et al. A novel enhancer regulates MGMT expression and promotes temozolomide resistance in glioblastoma. Nat Commun. 2018;9:2949. 30054476 10.1038/s41467-018-05373-4 PMC6063898

[20] Lin F, de Gooijer MC, Hanekamp D, Chandrasekaran G, Buil LC, Thota N, et al. PI3K-mTOR pathway inhibition exhibits efficacy against high-grade glioma in clinically relevant mouse models. Clin Cancer Res : J Am Assoc Cancer Res. 2017;23:1286–98. 10.1158/1078-0432.CCR-16-1276 27553832

[21] Xiao Y, Yu TJ, Xu Y, Ding R, Wang YP, Jiang YZ, et al. Emerging therapies in cancer metabolism. Cell Metab. 2023;35:1283–303. 37557070 10.1016/j.cmet.2023.07.006

[22] Wang T, Hu Q, Li B, Fan G, Jing D, Xu J, et al. Transcription factor EB reprograms branched-chain amino acid metabolism and promotes pancreatic cancer progression via transcriptional regulation of BCAT1. Cell Prolif. 2024;57:e13694. 38938061 10.1111/cpr.13694 PMC11533072

[23] Zheng Y, Zhu L, Qin ZY, Guo Y, Wang S, Xue M, et al. Modulation of cellular metabolism by protein crotonylation regulates pancreatic cancer progression. Cell Rep. 2023;42:112666. 37347667 10.1016/j.celrep.2023.112666

[24] Liu CC, Liu CC, Kanekiyo T, Xu H, Bu G. Apolipoprotein E and Alzheimer disease: risk, mechanisms and therapy. Nat Rev Neurol. 2013;9:106–18. 23296339 10.1038/nrneurol.2012.263 PMC3726719

[25] Tall AR, Yvan-Charvet L. Cholesterol, inflammation and innate immunity. Nat Rev Immunol. 2015;15:104–16. 25614320 10.1038/nri3793 PMC4669071

[26] Anand R, Prakash SS, Veeramanikandan R, Kirubakaran R. Association between apolipoprotein E genotype and cancer susceptibility: a meta-analysis. J cancer Res Clin Oncol. 2014;140:1075–85. 24706182 10.1007/s00432-014-1634-2 PMC11823783

[27] Pencheva N, Tran H, Buss C, Huh D, Drobnjak M, Busam K, et al. Convergent multi-miRNA targeting of ApoE drives LRP1/LRP8-dependent melanoma metastasis and angiogenesis. Cell. 2012;151:1068–82. 23142051 10.1016/j.cell.2012.10.028 PMC3753115

[28] Tavazoie MF, Pollack I, Tanqueco R, Ostendorf BN, Reis BS, Gonsalves FC, et al. LXR/ApoE activation restricts innate immune suppression in cancer. Cell. 2018;172:825–840.e818. 29336888 10.1016/j.cell.2017.12.026 PMC5846344

[29] Bancaro N, Calì B, Troiani M, Elia AR, Arzola RA, Attanasio G, et al. Apolipoprotein E induces pathogenic senescent-like myeloid cells in prostate cancer. Cancer cell. 2023;41:602–619.e611. 36868226 10.1016/j.ccell.2023.02.004

[30] Lou X, Ye Z, Xu X, Jiang M, Lu R, Jing D, et al. Establishment and characterization of the third non-functional human pancreatic neuroendocrine tumor cell line. Hum cell. 2022;35:1248–61. 35394261 10.1007/s13577-022-00696-3

[31] Xu J, Ye Z, Zhuo Q, Gao H, Qin Y, Llou X, et al. MEN1 degradation induced by neddylation and the CUL4B-DCAF7 axis promotes pancreatic neuroendocrine tumor progression. Cancer Res. 2023;83:2226–47. 36939378 10.1158/0008-5472.CAN-22-3599

[32] Liu M, Qin Y, Hu Q, Liu W, Ji S, Xu W, et al. SETD8 potentiates constitutive ERK1/2 activation via epigenetically silencing DUSP10 expression in pancreatic cancer. Cancer Lett. 2021;499:265–78. 33232789 10.1016/j.canlet.2020.11.023

[33] Denisenko E, de Kock L, Tan A, Beasley AB, Beilin M, Jones ME, et al. Spatial transcriptomics reveals discrete tumour microenvironments and autocrine loops within ovarian cancer subclones. Nat Commun. 2024;15:2860. 38570491 10.1038/s41467-024-47271-y PMC10991508

[34] Wang KJ, Wang C, Dai LH, Yang J, Huang H, Ma XJ, et al. Targeting an autocrine regulatory loop in cancer stem-like cells impairs the progression and chemotherapy resistance of bladder cancer. Clin Cancer Res. 2019;25:1070–86. 30397177 10.1158/1078-0432.CCR-18-0586

[35] Shi Y, Holtzman DM. Interplay between innate immunity and Alzheimer disease: APOE and TREM2 in the spotlight. Nat Rev Immunol. 2018;18:759–72. 30140051 10.1038/s41577-018-0051-1 PMC6425488

[36] Bergman PJ. Mechanisms of anticancer drug resistance. Vet Clin North Am Small Anim Pract. 2003;33:651–67. 12852241 10.1016/s0195-5616(03)00004-4

[37] Pegg AE. Multifaceted roles of alkyltransferase and related proteins in DNA repair, DNA damage, resistance to chemotherapy, and research tools. Chem Res Toxicol. 2011;24:618–39. 21466232 10.1021/tx200031q PMC3095683

[38] Knijnenburg TA, Wang L, Zimmermann MT, Chambwe N, Gao GF, Cherniack AD, et al. Genomic and molecular landscape of DNA damage repair deficiency across the cancer genome atlas. Cell Rep. 2018;23:239–254.e236. 29617664 10.1016/j.celrep.2018.03.076 PMC5961503

[39] Kass EM, Moynahan ME, Jasin M. When genome maintenance goes badly awry. Mol cell. 2016;62:777–87. 27259208 10.1016/j.molcel.2016.05.021 PMC4966655

[40] Moncharmont C, Levy A, Gilormini M, Bertrand G, Chargari C, Alphonse G, et al. Targeting a cornerstone of radiation resistance: cancer stem cell. Cancer Lett. 2012;322:139–47. 22459349 10.1016/j.canlet.2012.03.024

[41] Kishore C, Zi X. Wnt signaling and therapeutic resistance in castration-resistant prostate cancer. Curr Pharm Rep. 2023;9:261–74. 10.1007/s40495-023-00333-z PMC10664806 37994344

[42] Kim WK, Olson AW, Mi J, Wang J, Lee DH, Le V, et al. Aberrant androgen action in prostatic progenitor cells induces oncogenesis and tumor development through IGF1 and Wnt axes. Nat Commun. 2022;13:4364. 35902588 10.1038/s41467-022-32119-0 PMC9334353

[43] Ye YC, Zhao JL, Lu YT, Gao CC, Yang Y, Liang SQ, et al. NOTCH signaling via WNT regulates the proliferation of alternative, CCR2-independent tumor-associated macrophages in hepatocellular carcinoma. Cancer Res. 2019;79:4160–72. 31266773 10.1158/0008-5472.CAN-18-1691

[44] Demby T, Gross PS, Mandelblatt J, Huang JK, Rebeck GW. The chemotherapeutic agent doxorubicin induces brain senescence, with modulation by APOE genotype. Exp Neurol. 2024;371:114609. 37944881 10.1016/j.expneurol.2023.114609 PMC11302516

[45] Mulholland MM, Prinsloo S, Kvale E, Dula AN, Palesh O, Kesler SR. Behavioral and biologic characteristics of cancer-related cognitive impairment biotypes. Brain imaging Behav. 2023;17:320–8. 37127832 10.1007/s11682-023-00774-6 PMC10195718

[46] Ng CAS, Biran LP, Galvano E, Mandelblatt J, Vicini S, Rebeck GW. Chemotherapy promotes astrocytic response to Aβ deposition, but not Aβ levels, in a mouse model of amyloid and APOE. Neurobiol Dis. 2022;175:105915. 36336241 10.1016/j.nbd.2022.105915 PMC9794416

[47] Kemp SB, Carpenter ES, Steele NG, Donahue KL, Nwosu ZC, Pacheco A, et al. Apolipoprotein E promotes immune suppression in pancreatic cancer through NF-κB-mediated production of CXCL1. Cancer Res. 2021;81:4305–18. 34049975 10.1158/0008-5472.CAN-20-3929 PMC8445065

[48] Jayakar SK, Loudig O, Brandwein-Gensler M, Kim RS, Ow TJ, Ustun B, et al. Apolipoprotein E promotes invasion in oral squamous cell carcinoma. Am J Pathol. 2017;187:2259–72. 28751006 10.1016/j.ajpath.2017.06.016 PMC5762938

[49] Obradovic A, Chowdhury N, Haake SM, Ager C, Wang V, Vlahos L, et al. Single-cell protein activity analysis identifies recurrence-associated renal tumor macrophages. Cell. 2021;184:2988–3005.e2916. 34019793 10.1016/j.cell.2021.04.038 PMC8479759

[50] Wray J, Hartmann C. WNTing embryonic stem cells. Trends cell Biol. 2012;22:159–68. 22196214 10.1016/j.tcb.2011.11.004

[51] Hrckulak D, Kolar M, Strnad H, Korinek V. TCF/LEF transcription factors: an update from the internet resources. Cancers. 2016;8:70. 27447672 10.3390/cancers8070070 PMC4963812

[52] Alikhani N, Ferguson RD, Novosyadlyy R, Gallagher EJ, Scheinman EJ, Yakar S, et al. Mammary tumor growth and pulmonary metastasis are enhanced in a hyperlipidemic mouse model. Oncogene. 2013;32:961–7. 22469977 10.1038/onc.2012.113 PMC4063440

[53] Furuya H, Sakatani T, Tanaka S, Murakami K, Waldron RT, Hogrefe W, et al. Bladder cancer risk stratification with the Oncuria 10-plex bead-based urinalysis assay using three different Luminex xMAP instrumentation platforms. J Transl Med. 2024;22:8. 38167321 10.1186/s12967-023-04811-2 PMC10763405

[54] Li J, Gao A, Zhang F, Wang S, Wang J, Wang J, et al. ILT3 promotes tumor cell motility and angiogenesis in non-small cell lung cancer. Cancer Lett. 2021;501:263–76. 33152402 10.1016/j.canlet.2020.10.048

[55] Huang W, Li W, Chen X, Xiang C, Luo K. APOE drives glioma progression by modulating CCL5/CCR5 signaling in the tumor microenvironment and inducing M2 macrophage polarization. Immunobiology. 2025;230:152895. 40203505 10.1016/j.imbio.2025.152895

[56] Li Z, Li M, Sun S, Bin Y, Zuo S, Huo R, et al. APOE modulates ferroptosis to drive macrophage polarization toward the M2 type and enhance PTC migration and invasion. Immunobiology. 2025;230:152900. 40245754 10.1016/j.imbio.2025.152900

[57] Torres ID, Loureiro JA, Coelho MAN, Carmo Pereira M, Ramalho MJ. Drug delivery in glioblastoma therapy: a review on nanoparticles targeting MGMT-mediated resistance. Expert Opin Drug Deliv. 2022;19:1397–415. 36103209 10.1080/17425247.2022.2124967

[58] Ito M, Ohba S, Gaensler K, Ronen SM, Mukherjee J, Pieper RO. Early Chk1 phosphorylation is driven by temozolomide-induced, DNA double strand break- and mismatch repair-independent DNA damage. PloS one. 2013;8:e62351. 23667469 10.1371/journal.pone.0062351 PMC3646831

[59] Naumann SC, Roos WP, Jöst E, Belohlavek C, Lennerz V, Schmidt CW, et al. Temozolomide- and fotemustine-induced apoptosis in human malignant melanoma cells: response related to MGMT, MMR, DSBs, and p53. Br J Cancer. 2009;100:322–33. 19127257 10.1038/sj.bjc.6604856 PMC2634706

