# Supplementary material for: Tumor-derived apolipoprotein E confers resistance to temozolomide in pancreatic neuroendocrine tumors
Source: Cell Death Dis. 2025 Dec 13;17(1):35. doi: 10.1038/s41419-025-08317-1 (PMC12804833; doi:10.1038/s41419-025-08317-1)
Supplement: Supplementary file 1 — Figure S1-S6 [file 41419_2025_8317_MOESM1_ESM.docx]

**Tumor-derived Apolipoprotein E confers resistance to temozolomide in pancreatic neuroendocrine tumors**


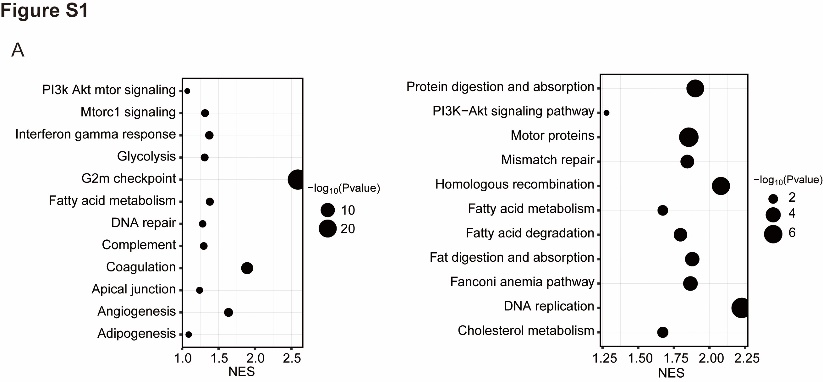


**Figure S1. High lipid metabolism confer resistance to TMZ in pNETs**

1. Based on transcriptome data, functional enrichment analysis in TMZ-treated cells.


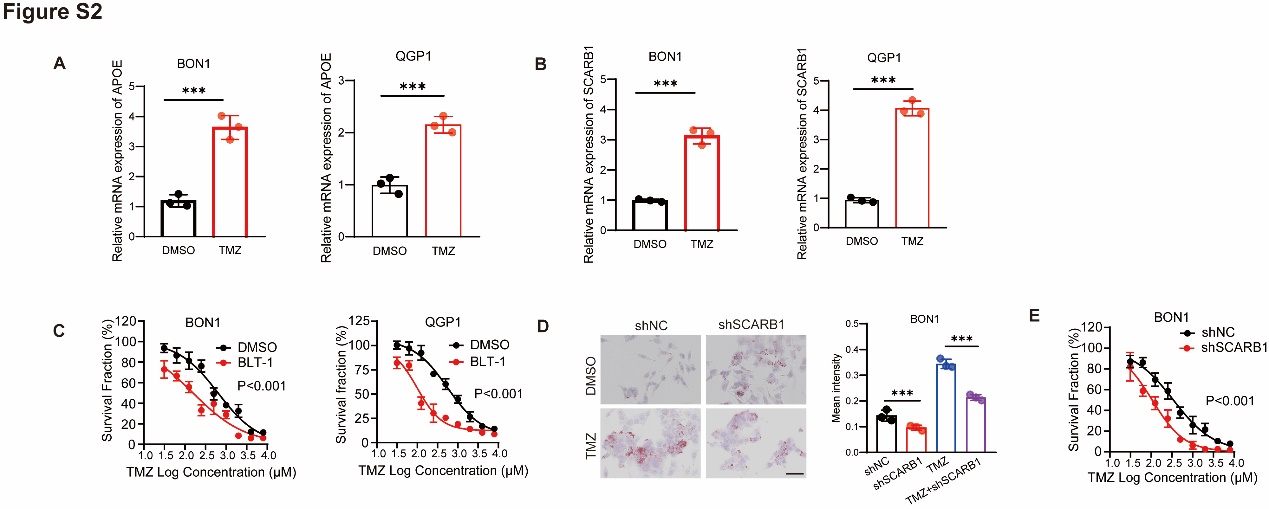


**Figure S2. Temozolomide promotes the secretion of APOE by pNETs to reshape lipid metabolism**

1. RT-qPCR revealed that APOE level in TMZ-treated BON1 and QGP1.
2. RT-qPCR revealed that SCARB1 level in TMZ-treated BON1 and QGP1.

**(C)** The effect of SCARB1 inhibitor BTL-1 on the cellular activity of BON1 and QGP1.

**(D)** Impact of SCARB1 knockdown on the change of TMZ-induced lipid droplets in BON1. Scale bar, 50μm.

**(E)** Impact of SCARB1 knockdown on BON1 sensitivity to TMZ.

Data in the graph are shown as the means ± SD from three independent experiment. * P<0.05, ** P<0.01, *** P<0.001, ns: no significance.


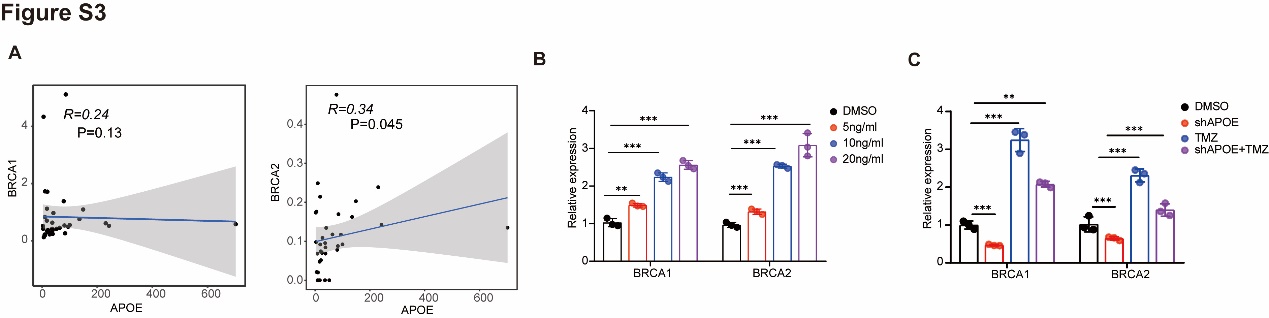


**Figure S3. APOE decreases TMZ sensitivity by Homologous recombination pathway**

**(A)** Demonstrate the correlation between APOE and BRCA1/2 based on transcriptomic data from cohort 2.

**(B)** Western blotting and RT-PCR experiments revealed that APOE enhances the expression of the HR genes BRCA1/2 in QGP1.

**(C)** Knocking down APOE decreased expression of the HR genes BRCA1/2 induced by TMZ in QGP1.


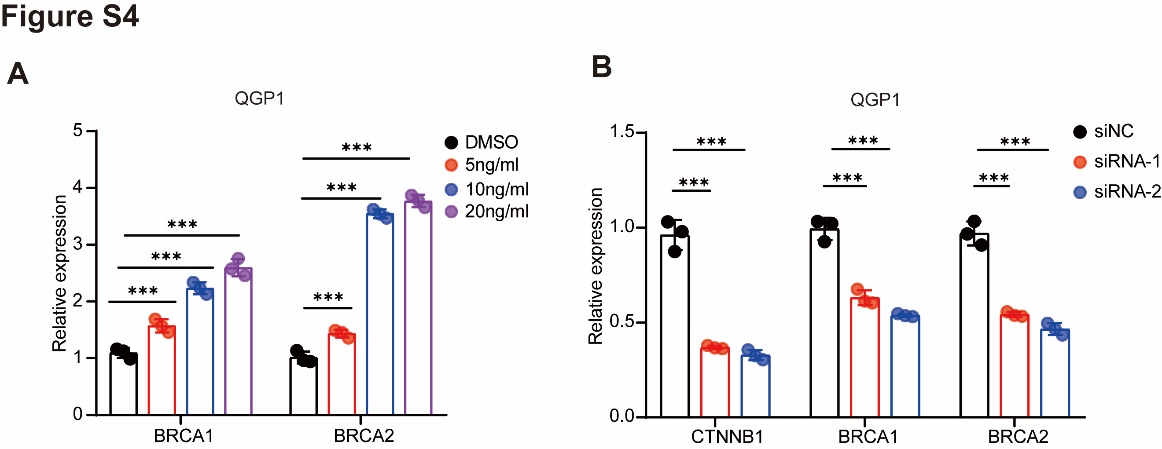


**Figure S4.** **APOE activated Wnt signal pathway and subsequent Wnt-induced DNA repair**

**(A)** PCR show APOE increase β-catenin accumulation in QGP1.

**(B)** PCR show knockdown of β-catenin by independent siRNAs inhibits HR genes BRCA1/2 in QGP1.


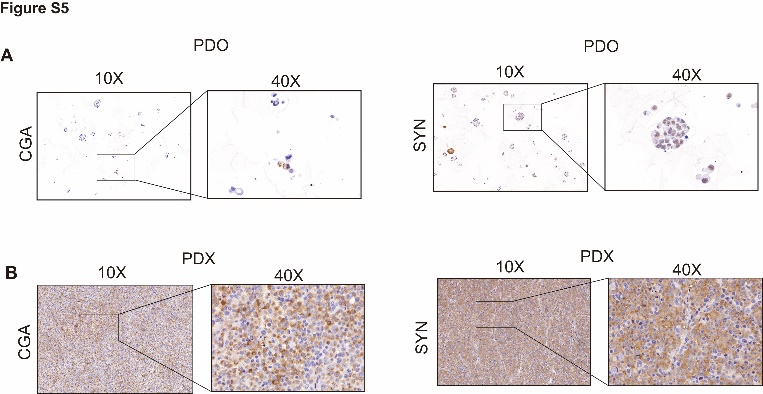


**Figure S5. The SCARB1 inhibitor BLT-1 increased TMZ sensitivity in pre-clinical models**

**(A-B)** The PDO and PDX also stained higher positive rate of neuroendocrine tumor indicators (CgA and SYN) on immunocytochemistry.


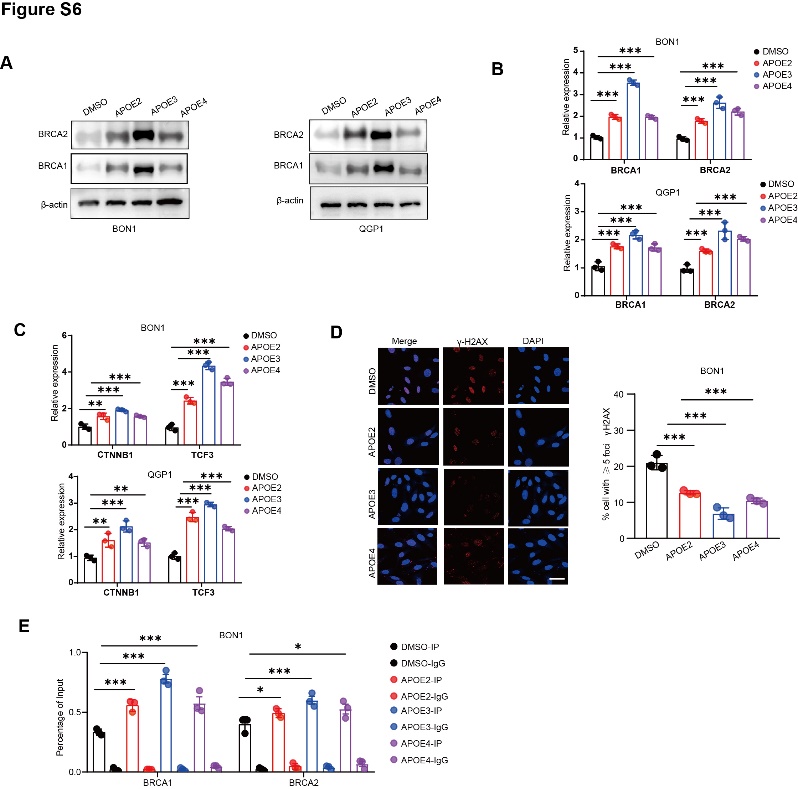


**Figure S6. The impact of different APOE genotypes on BRCA1/2-related DNA repair in tumor cells.**

**(A)** Western Blot showing the effects of treatment with different APOE genotypes on catenin and BRCA1/2 in BON1 and QGP1 cells. Cells were treated for 48 hours at a concentration of 10 ng/mL.

**(B–C)** Quantitative PCR analysis showing the effects of different APOE genotypes on the transcriptional expression of BRCA1/2 and CTNNB1/TCF3 in BON1 and QGP1 cells.

**(D)** Immunofluorescence assay demonstrating the influence of different APOE genotypes on DNA damage repair. Scale bar, 50μm.

**(E)** ChIP-PCR experiments evaluating the binding of TCF3 to three putative binding sites in BRCA1 and BRCA2 under different APOE isoform treatments.
